# Hemostatic effects of tranexamic acid in cardiac surgical patients with antiplatelet therapy: a systematic review and meta-analysis

**DOI:** 10.1186/s13741-024-00418-3

**Published:** 2024-06-17

**Authors:** Lijuan Tian, Xiaotao Li, Lixian He, Hongwen Ji, Yuntai Yao, the Evidence in Cardiovascular Anesthesia (EICA) Group

**Affiliations:** 1 Department of Anesthesiology, National Center for Cardiovascular Diseases, Fuwai Hospital, Chinese Academy of Medical Sciences and Peking Union Medical College, Beijing, 100037 China; 2 Department of Anesthesiology, Yunnan Fuwai Cardiovascular Hospital, Kunming, 650102 China

**Keywords:** Tranexamic acid, Cardiac surgery, Antiplatelet therapy, Post-operative bleeding

## Abstract

**Background:**

The purpose of the current study was to assess the efficacy of tranexamic acid (TXA) on reducing bleeding in cardiac surgical patients with preoperative antiplatelet therapy (APT).

**Methods:**

Five electronic databases were searched systematically for randomized-controlled trials (RCTs) assessing the impact of intravenous TXA on post-operative bleeding on cardiac surgical patients with preoperative APT until May 2024. Primary outcome of interest was post-operative blood loss. Secondary outcomes of interest included the incidence of reoperation due to post-operative bleeding, post-operative transfusion requirements of red blood cells (RBC), fresh-frozen plasma (FFP), and platelet concentrates. Mean difference (MD) with 95% confidence interval (CI) or odds ratios (OR) with 95% CI was employed to analyze the data. Subgroup and meta-regression analyses were performed to assess the possible influence of TXA administration on reducing bleeding and transfusion requirements.

**Results:**

A total of 12 RCTs with 3018 adult cardiac surgical patients (TXA group, 1510 patients; Control group, 1508 patients) were included. The current study demonstrated that TXA significantly reduced post-operative blood loss (*MD* =  − 0.38 L, 95% *CI*: − 0.73 to − 0.03, *P* = 0.03; *MD* =  − 0.26 L, 95% *CI*: − 0.28 to − 0.24, *P* < 0.00001; *MD* =  − 0.37 L, 95% *CI*: − 0.63 to − 0.10,* P* = 0.007) in patients receiving dual antiplatelet therapy (DAPT), aspirin, or clopidogrel, respectively. Patients in TXA group had significantly lower incidence of reoperation for bleeding as compared to those in Control group. The post-operative transfusion of RBC and FFP requirements was significantly lower in TXA group than Control group. Subgroup analyses showed that studies with DAPT discontinued on the day of surgery significantly increased the risk of post-operative blood loss [(*MD*: − 1.23 L; 95% *CI*: − 1.42 to − 1.04) vs. (*MD*: − 0.16 L; 95% *CI*: − 0.27 to − 0.05); *P* < 0.00001 for subgroup difference] and RBC transfusion [(*MD*: − 3.90 units; 95% *CI*: − 4.75 to − 3.05) vs. (*MD*: − 1.03 units; 95% *CI*: − 1.96 to − 0.10); *P* < 0.00001 for subgroup difference] than those with DAPT discontinued less than 5–7 days preoperatively.

**Conclusions:**

This meta-analysis demonstrated that TXA significantly reduced post-operative blood loss and transfusion requirements for cardiac surgical patients with preoperative APT. These potential clinical benefits may be greater in patients with aspirin and clopidogrel continued closer to the day of surgery.

**Trial registration number:**

CRD42022309427.

**Supplementary Information:**

The online version contains supplementary material available at 10.1186/s13741-024-00418-3.

## Introduction

Post-operative bleeding is a common and crucial clinical consequence of cardiac surgery with complex and multifactorial causes including procedures, patient characteristics, and medications (Paparella et al. 2004; Murphy et al. 2007). Most patients diagnosed with coronary artery disease receive preoperative dual antiplatelet therapy (DAPT) with aspirin and P2Y_12_ inhibitors to prevent occurrence of thrombotic events before cardiac surgery (Janssen et al. 2017). In elective surgery, discontinuation of antiplatelet therapy (APT) should be individually adjusted according to the consensus of anesthesiologists, surgeons, and patients to minimize ischemic risks and bleeding risks during surgery. Platelet dysfunction is one of the most common reasons for post-operative massive bleeding in cardiac surgical patients (Berger et al. 2012; Kremke et al. 2013). A national registry report from Sweden and a single-center study have demonstrated that transfusion of blood products was greater in patients with APT before coronary artery bypass grafting (CABG) surgery, and about 3.9% of mortality occurred in patients treated with clopidogrel (Tomšič et al. 2016; Hansson et al. 2016). To improve clinical prognosis, prevention of perioperative blood loss may be more effective than reducing allogeneic blood transfusions (Biancari et al. 2018). For patients at risk of bleeding, the current clinical practice guidelines strongly recommend the administration of antifibrinolytic agents to reduce blood loss and blood transfusion during cardiac surgery and blood conservation, based largely on results of large-scale clinical studies (Tibi et al. 2021; Pagano et al. 2018). Since aprotinin was withdrawn from the market, tranexamic acid (TXA) has become the most popular antifibrinolytic agent and provided effective benefit in antiplatelet-associated bleeding in cardiac surgery without significant thromboembolic events (Fischer et al. 2020a).

The effectiveness and efficacy of TXA on patients with impaired platelet dysfunction remain undetermined. Previous studies suggested that TXA administration could improve platelet function via inhibition of plasminogen to plasmin conversion (Pabinger et al. 2017; Mahla et al. 2018). Several studies have provided preliminary evidence that TXA could promote platelet function in patients undergoing CABG with APT and reduce post-operative bleeding and the incidence of allogeneic blood transfusion (Weber et al. 2011; Pleym et al. 2003). A prior review indicated that TXA administration was associated with a reduced risk of bleeding following either single antiplatelet treatment (SAPT) or DAPT by improving platelet function (Fischer et al. 2020b). Another recently published trial demonstrated that TXA administration was not associated with reduced blood loss volume in patients undergoing major operations (Amour et al. 2016).

The purpose of the present study was to evaluate the blood-saving effects of TXA administration on adult cardiac surgical patients with preoperative APT.

## Methods

### Ethics

According to the Ethical Committee of Fuwai Hospital, ethics approval was not required for meta-analysis. The protocol of current meta-analysis has been registered on the International Prospective Systematic Reviews Registry database (CRD42022309427).

### Patient and public involvement

Patients and/or the public did not participate in the design, or execute, or statement, or dissemination of this study.

### Search strategy

We conducted a systemic review according to Preferred Reporting Items for Systematic Reviews and Meta-Analysis Guidelines (PRIMSA) (Higgins et al. 2020) (Supplemental Table 1, PRISMA Checklist). PubMed, Ovid, Embase, the Cochrane Central Register of Controlled Trials (CENTRAL), and China National Knowledge Infrastructure (CNKI) database (until May 2024) were systematically searched to all RCTs assessing the impact of intravenous TXA administration on post-operative bleeding and allogeneic blood products for adult cardiac surgical patients with preoperative APT. The searching keywords used were as follows: (cardiac surgery OR coronary artery bypass surgery OR cardiovascular surgery OR operation OR procedure) AND (aspirin OR clopidogrel OR antiplatelet OR single antiplatelet therapy OR dual antiplatelet therapy OR SAPT OR DAPT OR acetylsalicylic acid OR ticagrelor) AND (tranexamic acid) AND (randomized controlled trial OR controlled clinical trial OR randomized OR placebo OR randomly OR trial). Detailed search strategies were reported in Supplemental Table 2. No language restriction was applied. To make sure that all relevant articles were included, references of highlighted research were thoroughly searched. Two authors (L. J. T. and Y. T. Y.) independently reviewed all the retrieved records by reading the titles, abstracts, and keywords in a non-blind, standardized manner to determine which studies met the inclusion criteria and removed those ineligible ones. The eligibility studies ultimately included in the analysis were further determined by evaluating the full text. Any discrepancy was resolved through discussion, and other reviewers (X. T. L. and L. X. H.) participated to resolve conflicts.

**Table 1:** Characteristics of included studies

Author, year	Country	Surgery	Sample size	Group	TXA group	Control group	Antiplatelet medication	Outcome
(Ahn et al. 2012)	Korea	OPCAB	76	2	TXA (*n* = 38): 1 g before SI, 200 mg/h until OP end	Saline (*n* = 38)	Stop clopidogrel and aspirin less than 5 days preoperatively	①, ②, ③, ④, ⑧
(Shi et al. 2013a) (1)	China	On-pump CABG	117	2	TXA (*n* = 58): 15 mg/kg before SI, 15 mg/kg after protamine neutralization	Saline (*n* = 59)	Stop clopidogrel and aspirin less than 7 days preoperatively	①, ②, ③, ④, ⑤, ⑥, ⑦, ⑧
(Shi et al. 2013b) (2)	China	On-pump CABG	110	2	TXA (*n* = 55): 10 mg/kg after induction, 10 mg/kg/h until OP end	Saline (*n* = 55)	Stop clopidogrel and aspirin less than 7 days preoperatively	①, ②, ③, ④, ⑤, ⑥, ⑦, ⑧
(Altun et al. 2017)	Turkey	On-pump CABG	54	4	TXA (*n* = 18): 10 mg/kg before SI, continued 1 mg/kg/h for 10 h	①Blank (*n* = 10): No drug ②TXA + Des (*n* = 16): 10 mg/kg before SI, 1 mg/kg/h for 10 h, Des 0.3 µg/kg after protamine neutralization ③Des (*n* = 10): 0.3 µg/kg after protamine neutralization	Stop clopidogrel and aspirin until the day of surgery	①, ②, ③, ④, ⑤, ⑥, ⑦
(Banihashem et al. 2019)	Iran	On-pump CABG	120	2	TXA (*n* = 60): 10 mg/kg before SI, 10 mg/kg after protamine neutralization	Saline (*n* = 60)	Stop clopidogrel and aspirin less than 5 days preoperatively	①, ②, ④, ⑤, ⑥
(Khadanga et al. 2020)	India	OPCAB	60	2	TXA (*n* = 30): 10 mg/kg after induction	Saline (*n* = 30)	Stop clopidogrel 5 days preoperatively, aspirin until the day of surgery	①, ⑤, ⑥, ⑦, ⑧
(Landymore et al. 1997)	Canada	On-pump CABG	198	4	TXA (*n* = 56): 10 mg/kg before CPB, continued 1 mg/kg/h during CPB	①Blank (*n* = 50): No drug ②Aprotinin (*n* = 48): 20 × 10^4^ KIU before CPB, 20 × 10^4^ KIU /h during CPB ③EACA (*n* = 44): 5 g before CPB, 1 g/h during CPB	Stop aspirin 48 h preoperatively	①
(Pleym et al. 2003)	Norway	On-pump CABG	79	2	TXA (*n* = 40): 30 mg/kg before CPB	Saline (*n* = 39)	Stop aspirin until the day of surgery	①, ②, ③, ④, ⑧
(Guo et al. 2007)	China	OPCAB	112	3	TXA (*n* = 36): 0.75 g before SI, 0.25 g/h until OP end	①Saline (*n* = 40) ②Aprotinin (*n* = 36): 10 × 10^5^ KIU before SI, 5 × 10^5^ KIU/h until OP end	Stop aspirin 5–7 days preoperatively	①, ②, ③
(Van Aelbrouck et al. 2016)	Belgium, USA	Cardiac surgery	28	4	①TXA1 (*n* = 9): Aspirin discontinued before the day of surgery with TXA 30 mg/kg loading dose followed by 16 mg/kg/h until OP end ②TXA2 (*n* = 5): No aspirin patients with TXA 30 mg/kg loading dose followed by 16 mg/kg/h until OP end	①Saline1 (*n* = 9): Aspirin discontinuation before the day of surgery treated with saline ②Saline2 (*n* = 5): No aspirin patients treated with saline	Stop aspirin until the day of surgery	①, ②, ③, ④
(Myles et al. 2017)	Australian, Canada, Italy, Netherlands, New Zealand, China (Hong Kong), UK	OPCAB and on-pump CABG	4662	4	①TXA1 (*n* = 1047): Aspirin discontinuation before the day of surgery with TXA 100 mg/kg or 50 mg/kg after induction ②TXA2 (*n* = 1264): No aspirin patients treated with TXA 100 mg/kg or 50 mg/kg after induction	①Saline1 (*n* = 1053): Aspirin discontinuation before the day of surgery treated with saline ②Saline2 (*n* = 1267): No aspirin patients treated with saline	Stop aspirin until the day of surgery	①, ②, ③, ⑧
(Shi et al. 2013c) (3)	China	On-pump CABG	552	6	①TXA1 (*n* = 63): Clopidogrel discontinued within 7 days before surgery with TXA 10 mg/kg after induction, 10 mg/kg/h until OP end ②TXA2 (*n* = 105): Clopidogrel discontinued for more than 7 days with TXA 10 mg/kg after induction, 10 mg/kg/h until OP end ③TXA3 (*n* = 106): No clopidogrel patients with TXA 10 mg/kg after induction, 10 mg/kg/h until OP end	①Saline1 (*n* = 65): Clopidogrel discontinued within 7 days before surgery with saline ②Saline2 (*n* = 106): Clopidogrel discontinued for more than 7 days with saline ③Saline3 (*n* = 107): No clopidogrel patients with saline	Stop clopidogrel less than 7 days preoperatively	①, ②, ③, ④, ⑧

*OPCAB* Off-pump coronary artery bypass graft, *CABG* Coronary artery bypass grafting, *TXA* Tranexamic acid, *SI* Skin incision, *OP* Operation, *CPB* Cardiopulmonary bypass, *Des* Desmopressin acetate, *EACA* Aminocaproic acid. Outcomes ①, bleeding; ②, post-operative red blood cell transfusion rate and volume; ③, post-operative fresh-frozen plasma transfusion rate and volume; ④, post-operative platelet concentrates transfusion rate and volume; ⑤, mechanical ventilation duration; ⑥, lengths of stay in the intensive care unit; ⑦, hospital length of stay; ⑧, reoperation for bleeding

### Inclusion and exclusion criteria

The inclusion criteria were as follows: (1) adult cardiac surgical patients with preoperative APT, (2) intraoperative intravenous TXA administration versus placebo or blank, (3) randomized controlled trials (RCTs), and (4) at least one of the predetermined outcomes listed in the following reported. Primary outcomes were post-operative blood loss. Secondary outcomes were the incidence of reoperation due to post-operative bleeding, post-operative transfusion of red blood cells (RBC), fresh-frozen plasma (FFP) and platelet concentrates (PC), and post-operative recovery including mechanical ventilation duration (MVD), and post-operative length of stay (LOS) in the intensive care unit and hospital.

Exclusion criteria included the following: (1) studies published as review articles, case reports, expert experience, or abstracts, (2) retrospective or observational studies, (3) studies based on animal models, (4) outcomes of interest could not be extracted and analyzed, (5) duplicate publications, and (6) aminocaproic acid or aprotinin as control.

### Study quality assessment

Two investigators (L. J. T. and Y. T. Y.) independently assessed the risk of bias by using the tool described in the Cochrane Handbook for Systematic Reviews of Interventions (Higgins et al. 2011). The quality of the study was categorized as low risk if there is no indication of risk of bias, medium risk if there is a potential risk of bias, or high risk if there is a clear indication of risk of bias. Additionally, L. J. T. and Y. T. Y. independently evaluated the methodologic quality of each included trial using the modified Jadad score (Jadad et al. 1996). Modified Jadad quality scoring scale included the generation of random sequences, randomized concealment, and whether blind method and reporting the withdrawals was adopted. For each item, there were associated criteria and scores, with less than or equal to 3 points as low-quality research and 4–7 points as high-quality research.

### Data abstraction

General information, participants characteristics, intervention, and follow-up data for eligible study were independently extracted by two investigators L. J. T. and Y. T. Y. and included research title, first author, year of publication, journal, country, number of patients, gender, age, type of surgical procedure, and data regarding outcomes of interest. Discrepancies were discussed among all authors at the end of assessment.

### Statistical analysis

Statistical analyses were performed using Review Manager 5.4 (Cochrane Collaboration, Oxford, UK) and STATA 12.0 (Stata Corp., College Station, TX, USA). Continuous variables and treatment effect were presented as mean difference (MD) with 95% confidence interval (CI). Dichotomous variables analyzed with odds ratio (OR) with 95% CI. Additionally, the formulas of (Wan et al. 2014) transformed continuous variables that were described as median and interquartile range (IQR) into mean and standard deviation (SD). The random-effect model was used to pool the data for the consideration of methodological and clinical heterogeneity (*I*^2^ > 50% or *P* < 0.05), and the fixed-effect model was used for analysis that there was no significant heterogeneity. Statistical heterogeneity among studies was assessed using the Q test and *I*^2^ statistics. According to the Cochrane Handbook, the percentages of *I*^2^ at 0–25%, 25–50%, and 75–100% indicate low, medium, and high heterogeneity, respectively. After statistical heterogeneity is established, the researchers searched for possible sources from the clinical and methodological perspective and then perform subgroup or sensitivity analysis to detect the possible causes of heterogeneity. The sensitivity analysis was performed to evaluate the influence of individual study on the overall estimate by omitting each study in turn. Subgroup and meta-regression analyses were performed to detect the possible sources of inconsistency and heterogeneity. Meta-regression was conducted with the following covariates: (I) SAPT and DAPT and (II) TXA regimens. Subgroup analysis was employed to investigate the association between different DAPT discontinued time and surgical technology and clinical outcomes (post-operative bleeding and allogeneic blood transfusion). Publication biases were examined with the Begg’s test and visualized the symmetry of the funnel plots of the outcomes (Egger et al. 1997). All *P*-values were two sided, and statistical significance was defined as *P* < 0.05.

## Results

### Literature search, study characteristics, and quality assessment

**Fig. 1:**
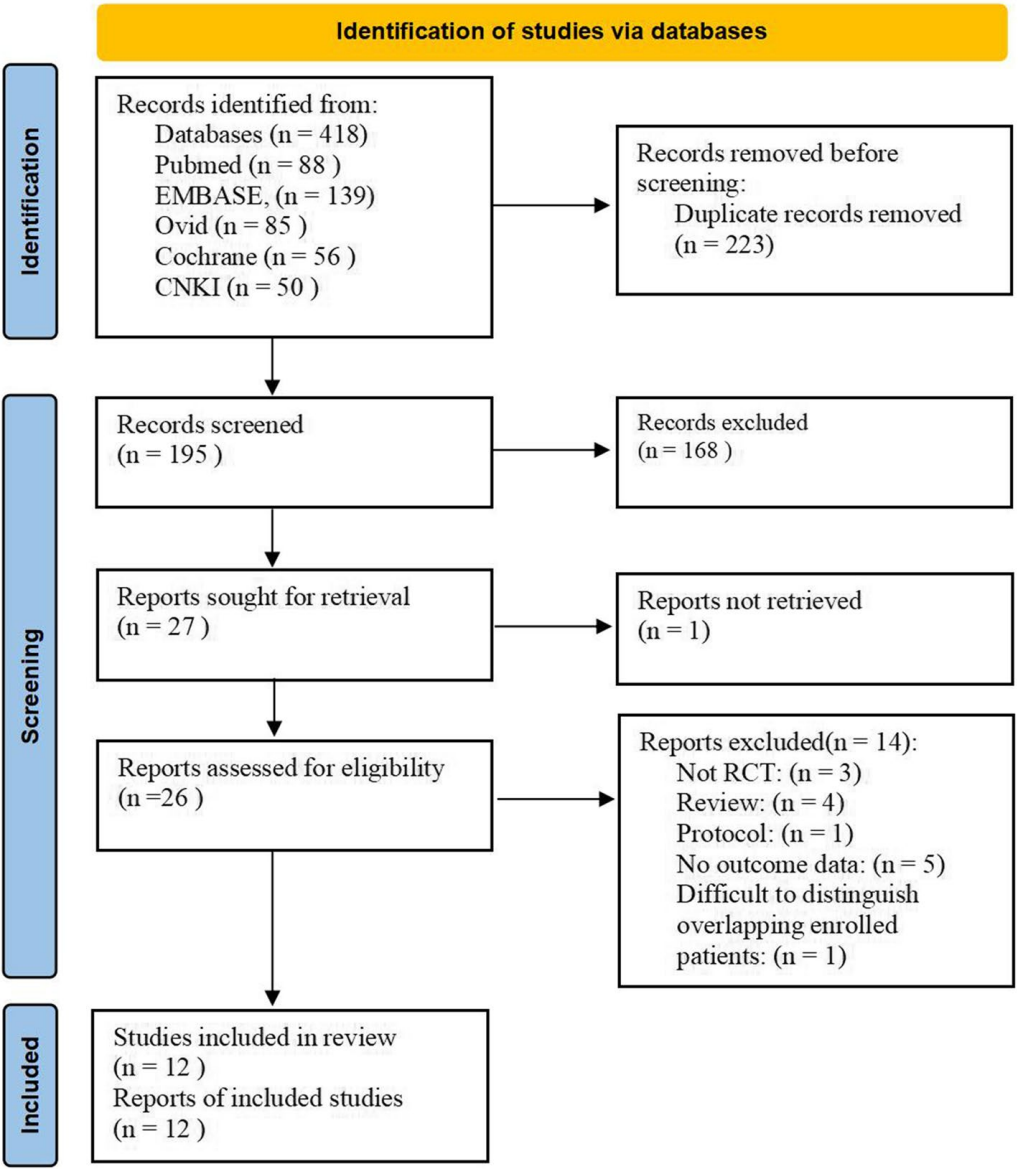
Flowchart of the study search and selection process

As shown in the flowchart (Fig. 1), 418 records were initially identified in the databases. A total of 392 trials were excluded due to duplication and review of titles and abstracts. Finally, 12 (Pleym et al. 2003; Ahn et al. 2012; Shi et al. 2013a, 2013b, 2013c; Altun et al. 2017; Banihashem et al. 2019; Khadanga et al. 2020; Landymore et al. 1997; Guo et al. 2007; Aelbrouck et al. 2016; Myles et al. 2017) eligible trials that satisfied the inclusion criteria were included in this meta-analysis. Descriptive analyses of these articles were presented in Table 1. Of the 12 eligible trials, 2 (Shi et al. 2013b; Guo et al. 2007) were written in Chinese, the other 10 (Pleym et al. 2003; Ahn et al. 2012; Shi et al. 2013a, 2013c; Altun et al. 2017; Banihashem et al. 2019; Khadanga et al. 2020; Landymore et al. 1997; Aelbrouck et al. 2016; Myles et al. 2017) were in English (1 (Ahn et al. 2012) from Korea, 2 (Shi et al. 2013a, 2013c) from China, 1 (Altun et al. 2017) from Turkey, 1 (Banihashem et al. 2019) from Iran, 1 (Khadanga et al. 2020) from India, 1 (Landymore et al. 1997) from Canada, 1 (Pleym et al. 2003) from Norway, 1 (Aelbrouck et al. 2016) performed in Belgium and USA, 1 (Myles et al. 2017) performed in Australia, Canada, Italy, the Netherlands, New Zealand, China (Hong Kong), and UK). Seven trials (Pleym et al. 2003; Shi et al. 2013a, 2013b, 2013c; Altun et al. 2017; Banihashem et al. 2019; Landymore et al. 1997) included participants undergoing on-pump CABG, three trials (Ahn et al. 2012; Khadanga et al. 2020; Guo et al. 2007) included off-pump CABG patients, and two trials (Aelbrouck et al. 2016; Myles et al. 2017) included mixed cardiac surgical patients.

### Study patient and intervention characteristics

The characteristics of the 12 RCTs are presented in Table 1. The screening included 3018 patients undergoing cardiac surgery, and 1510 were assigned to TXA group and 1508 to Control group. Eleven trials compared TXA with saline (Pleym et al. 2003; Ahn et al. 2012; Shi et al. 2013a, 2013b, 2013c; Altun et al. 2017; Banihashem et al. 2019; Khadanga et al. 2020; Guo et al. 2007; Aelbrouck et al. 2016; Myles et al. 2017), and 1 trial compared TXA with blank (Landymore et al. 1997). Six trials (511 patients), 5 trials (2379 patients), and 1 trial (128 patients) described patients who underwent DAPT, aspirin, or clopidogrel therapy, respectively. TXA administration regimens (dosage, timing of administration, and route) were not uniform due to differences in the design endpoints of the included trials. The loading dose of TXA ranged from 10 to 100 mg/kg and maintaining dose from 0 to 16 mg/kg/h, respectively.

### Effects on post-operative blood loss volume

**Fig. 2:**
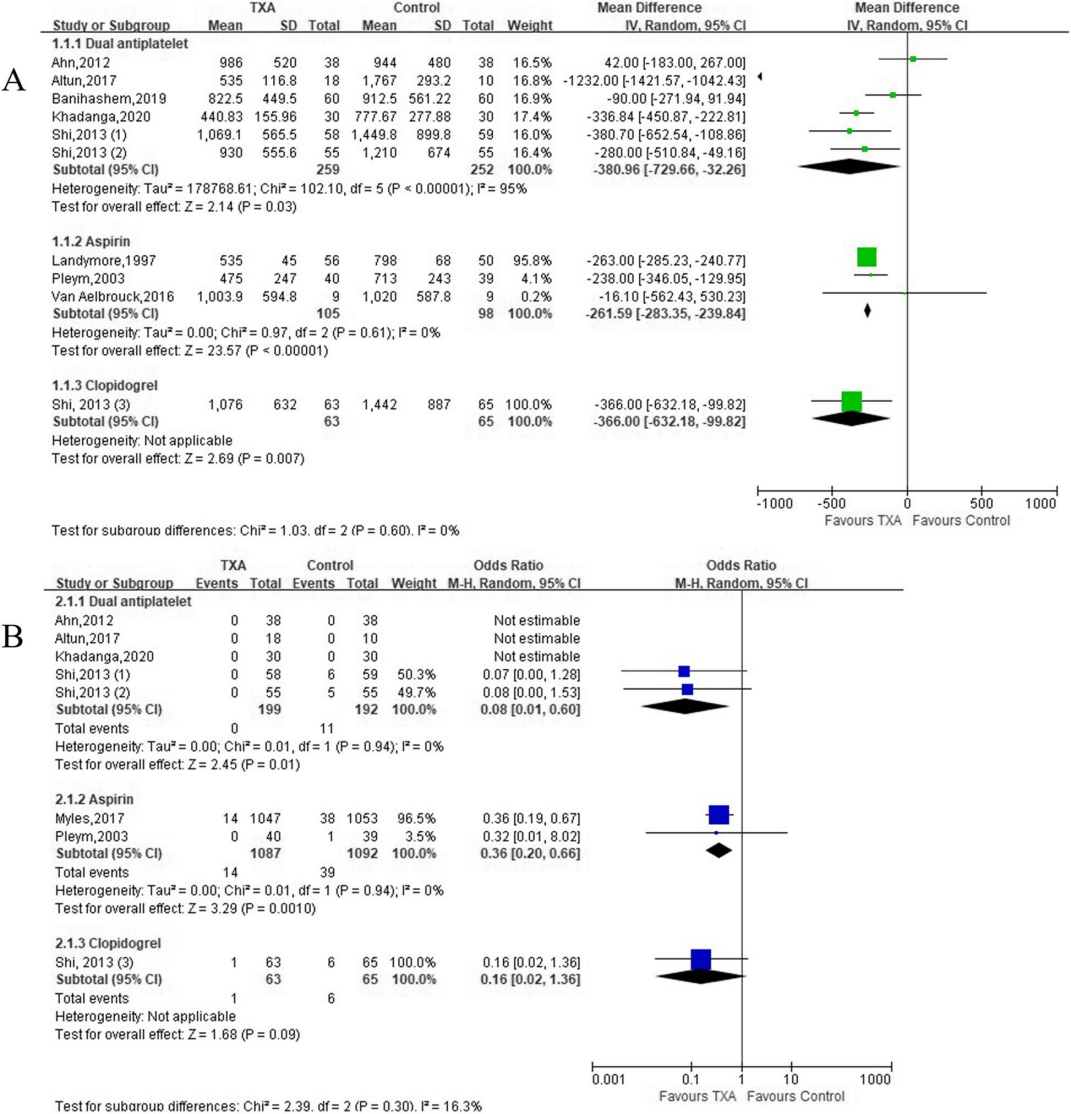
Forest plot of **A** post-operative bleeding volume and **B** the incidence of reoperation for bleeding

Six trials (6 comparisons, 511 patients), 3 trials (3 comparisons, 203 patients), and 1 trial (one comparison, 128 patients) described post-operative blood loss volume in patients with DAPT, aspirin, and clopidogrel, respectively (Table 1). Meta-analysis revealed that TXA significantly reduced post-operative blood loss volume in patients receiving DAPT [(*MD* =  − 0.38 L; 95% *CI*: − 0.73 to − 0.03; *P* = 0.03) with heterogeneity (*I*^2^ = 95%, *P* < 0.00001)], aspirin [(*MD* =  − 0.26 L; 95% *CI*: − 0.28 to − 0.24; *P* < 0.00001) without heterogeneity (*I*^2^ = 0%, *P* < 0.61)], and clopidogrel (*MD* =  − 0.37 L; 95% *CI*: − 0.63 to − 0.10; *P* = 0.007) (Fig. 2A).

### Effects on reoperation for bleeding

Eight trials (2698 patients) reported reoperation for bleeding with an overall incidence of 2.63% (TXA group, 1.11% vs Control group, 4.15%) (Table 1). Compared with Control group, there was a significant reduction in the incidence of reoperation for bleeding in patients receiving DAPT (*OR* = 0.08; 95% *CI*: 0.01 to 0.60; *P* = 0.01) without heterogeneity, aspirin (*OR* = 0.36; 95% *CI*: 0.20 to 0.66; *P* = 0.001) without heterogeneity, and clopidogrel (*OR* = 0.16; 95% *CI*: 0.02 to 1.36; *P* = 0.01) (Fig. 2B).

### Effects on post-operative RBC transfusion

**Fig. 3:**
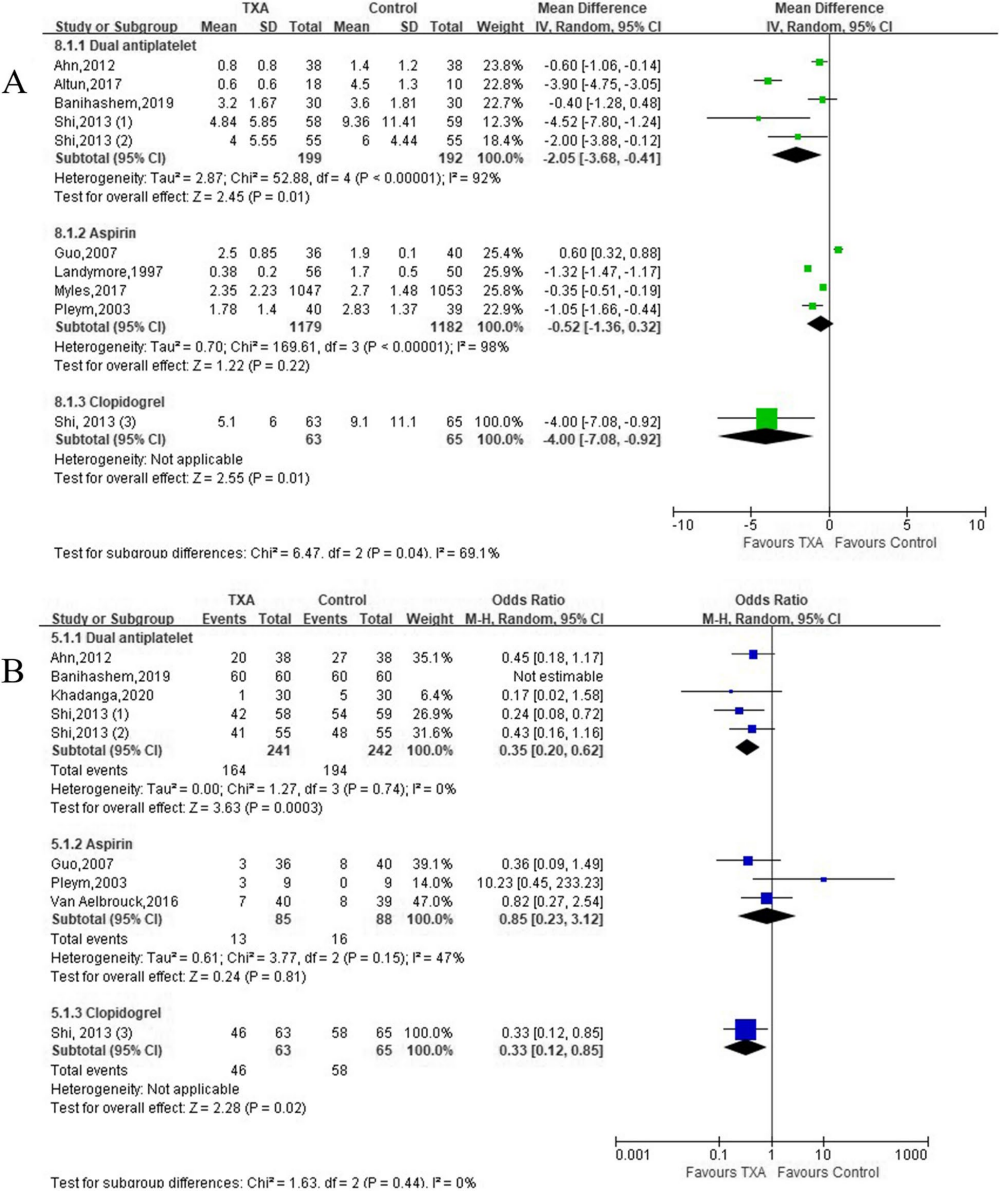
Forest plot of **A** RBC transfusion volume and **B** transfusion rate

Five trials (5 comparisons, 391 patients), 4 trials (4 comparisons, 2361 patients), and 1 trial (1 comparison, 128 patients) described post-operative RBC transfusion volume in patients with DAPT, aspirin, and clopidogrel, respectively (Table 1). Meta-analysis demonstrated that TXA significantly reduced post-operative RBC transfusion in patients receiving DAPT [(*MD* =  − 2.05 units; 95% *CI*: − 3.68 to − 0.41; *P* = 0.01) with heterogeneity (*I*^2^ = 92%, *P* < 0.00001)], aspirin [(*MD* =  − 0.52 units; 95% *CI*: − 1.36 to 0.32; *P* = 0.22) with heterogeneity (*I*^2^ = 98%, *P* < 0.00001)], and clopidogrel (*MD* =  − 4.00 units; 95% *CI*: − 7.08 to − 0.92; *P* = 0.01) (Fig. 3A). Nine trials with 784 patients reported post-operative RBC transfusion rate (TXA group, 57.33% vs. Control group, 67.85%). Compared with Control group, there was a significant reduction in the incidence of post-operative RBC transfusion in patients receiving DAPT (*OR* = 0.35; 95% *CI*: 0.20 to 0.62; *P* = 0.0003) without heterogeneity and clopidogrel (*OR* = 0.33; 95% *CI*: 0.12 to 0.85; *P* = 0.02) (Fig. 3B).

### Effects on post-operative FFP transfusion

**Fig. 4:**
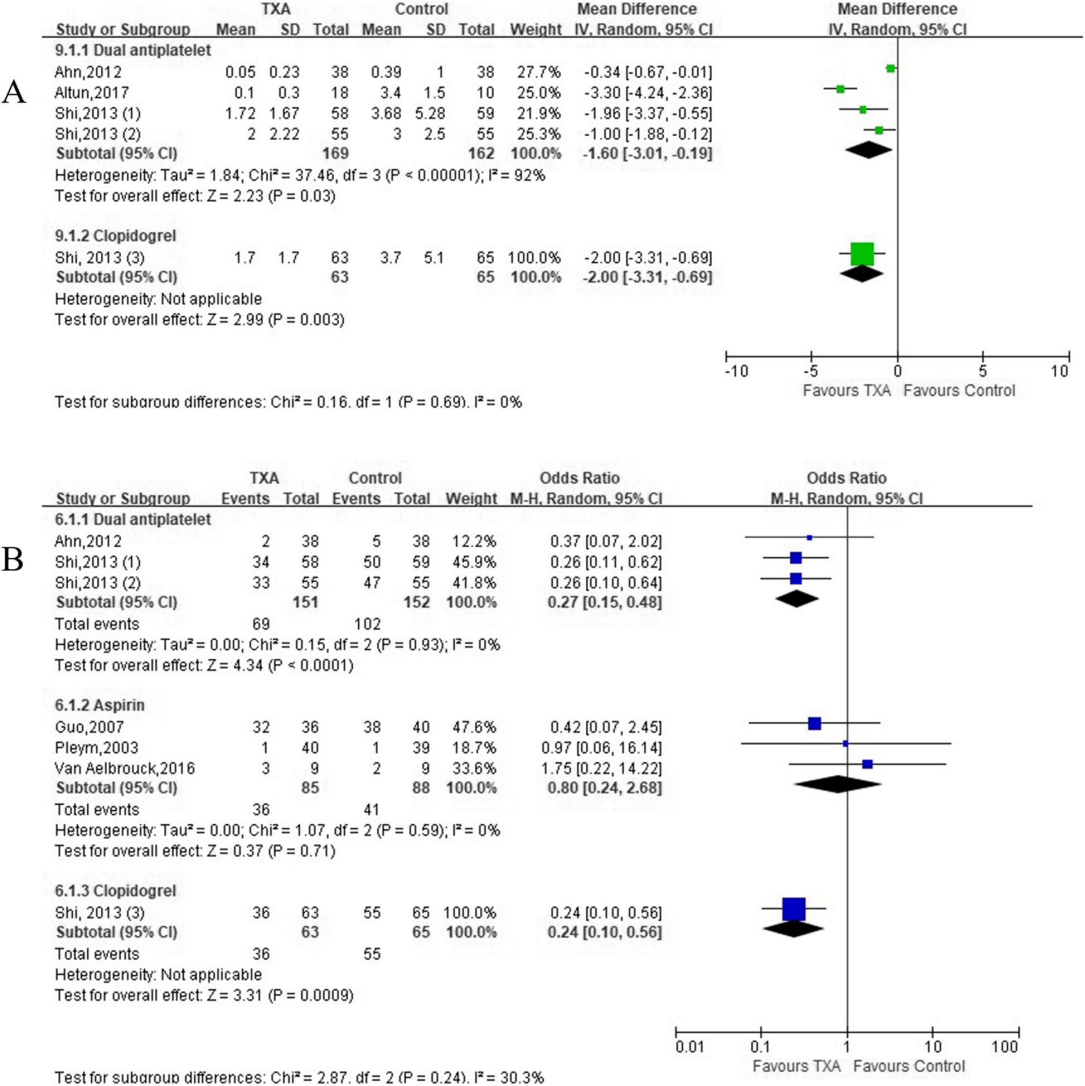
Forest plot of **A** FFP transfusion volume and **B** transfusion rate

Four trials (4 comparisons, 331 patients) and 1 trial (1 comparison, 128 patients) reported post-operative FFP transfusion volume in patients with DAPT and clopidogrel, respectively (Table 1). TXA significantly reduced post-operative FFP transfusion volume in patients receiving DAPT [(*MD* =  − 1.60 units; 95% *CI*: − 3.01 to − 0.19; *P* = 0.03) with heterogeneity (*I*^2^ = 92%, *P* < 0.00001)], and clopidogrel [(*MD* =  − 2.00 units; 95% *CI*: − 3.31 to − 0.69; *P* = 0.003) (Fig. 4A). Seven trials with 604 patients described post-operative FFP transfusion rate (TXA group, 47.16% vs. Control group, 64.92%). Meta-analysis demonstrated that there was a significant reduction in the incidence of post-operative FFP transfusion in patients receiving DAPT (*OR* = 0.27; 95% *CI*: 0.15 to 0.48; *P* < 0.0001) without heterogeneity and clopidogrel (*OR* = 0.24; 95% *CI*: 0.10 to 0.56; *P* = 0.0009) in TXA group (Fig. 4B).

### Effects on post-operative PC transfusion

Three trials (3 comparisons, 265 patients) in patients with DAPT reported post-operative PC transfusion volume in patients with DAPT (Table 1). The meta-analysis demonstrated that PC transfusion volume was similar between the TXA group and Control group (*MD* =  − 0.05 units; 95% *CI*: − 0.52 to 0.42; *P* = 0.83) without heterogeneity (Supplemental Fig. 3A). Three trials (3 comparisons, 303 patients) and 2 trials (2 comparisons, 97 patients) reported post-operative PC transfusion rate in patients with DAPT and aspirin, respectively (Table 1). Meta-analysis demonstrated that PC transfusion rate was no significant different between the two groups (6% vs. 6.5%) without heterogeneity (Supplemental Fig. 3B).

### Effects on post-operative recovery

The post-operative MVD was explored in 5 trials including 435 patients receiving DAPT without statistically significant difference between the 2 groups (Table 1, Supplemental Fig. 4A). The LOS in ICU (5 comparisons, 435 patients) and hospital (4 comparisons, 315 patients) were examined in cardiac surgical patients with DAPT (Table 1). The post-operative LOS in ICU was similar between the TXA group and Control group (Supplemental Fig. 4B). The length of stay in hospital was comparable to no significant difference between the two groups (Supplemental Fig. 4C).

### Meta-regression and subgroup analyses for potential sources of heterogeneity

The APT schemes (SAPT or DAPT) and TXA regimens (bolus or bolus plus continuous infusion) were included in the univariate meta-regression analyses for the post-operative blood loss volume in all studies. Results from the analysis of 10 studies including 842 patients showed no association of APT schemes (coefficient =  − 1.52, 95% *CI*: − 4.05 to 1.01; *P* = 0.20) or TXA regimens (coefficient =  − 1.23, 95% *CI*: − 4.79 to 2.33; *P* = 0.45) with TXA reducing post-operative blood loss.

Subgroup analyses showed that studies with DAPT discontinued on the day of surgery significantly increased the risk of post-operative blood loss volume [(*MD*: − 1.23 L; 95% *CI*: − 1.42 to − 1.04) vs. (*MD*: − 0.16 L; 95% *CI*: − 0.27 to − 0.05); *P* < 0.00001 for subgroup difference] and post-operative RBC transfusion volume [(*MD*: − 3.90 units; 95% *CI*: − 4.75 to − 3.05) vs. (*MD*: − 1.03 units; 95% *CI*: − 1.96 to − 0.10); *P* < 0.00001 for subgroup difference] than those with DAPT discontinued less than 5–7 days preoperatively. There was no significant difference in the risk of increased post-operative blood loss, RBC, and FFP transfusion volume for cardiac surgical patients with CPB or not (Supplemental Table 3).

### Quality assessment

Five studies with an unclear risk of bias were due to the unclear study design in detail for the selective reporting bias (Pleym et al. 2003; Ahn et al. 2012; Landymore et al. 1997; Guo et al. 2007; Aelbrouck et al. 2016). Three trials failed to report information on random sequence generation and were rated to have unclear risk of bias for this item (Altun et al. 2017; Landymore et al. 1997; Guo et al. 2007). Two trials were classified high risk of blinding assessments or participants and personnel (Altun et al. 2017; Khadanga et al. 2020), one trial was classified high risk of random sequence generation (Banihashem et al. 2019), and the other RCTs were assessed as low bias risk, indicating that they were of good quality (Supplemental Fig. 1 and Fig. 2). Of the 12 included trials, 9 trials (Pleym et al. 2003; Ahn et al. 2012; Shi et al. 2013a, 2013b, 2013c; Banihashem et al. 2019; Guo et al. 2007; Aelbrouck et al. 2016; Myles et al. 2017) had modified Jadad scores ≥ 4 and were considered as high-quality RCTs (Supplemental Table 4).

### Sensitivity analyses and publication bias

Sensitivity analysis was performed by removal of each study to analyze the influence of the overall treatment effect on high heterogeneity outcomes (Supplemental Table 5), whereas no contradictory results were found. For the reduction of post-operative blood loss volume and FFP transfusion volume in patients receiving DAPT, heterogeneity changed from high to low by exclusion of two studies conducted from Ahn et al. (patients underwent OPCAB) (Ahn et al. 2012) and Altun et al. (preoperative DAPT discontinued on the day of surgery) (Altun et al. 2017). For the reduction of post-operative RBC transfusion volume, heterogeneity changed from 95 to 14% for patients receiving DAPT by exclusion of two studies conducted from Shi et al. (patients with 15 mg/kg TXA after protamine neutralization) (Shi et al. 2013a) and Altun et al. (preoperative DAPT discontinued on the day of surgery) (Altun et al. 2017), and 98% to 0 for patients receiving aspirin by exclusion of two studies conducted from Guo et al. (patients underwent OPCAB) (Guo et al. 2007) and Myles et al. (patients underwent OPCAB or on-pump CABG) (Myles et al. 2017). No significant publication bias was detected by funnels plot examination with respect to post-operative bleeding volume (Supplemental Fig. 5). *P*-values for the Begg tests were > 0.05, suggesting a low probability of publication bias. It suggested that there was no obvious publication bias in post-operative bleeding volume (Begg’s *P* = 0.13).

## Discussion

The current study is the first of its kind to systemically determine the efficacy of tranexamic acid on post-operative bleeding and transfusion requirements for adult cardiac surgical patients with preoperative APT. TXA administration reduced post-operative bleeding in patients with DAPT or aspirin, RBC, and FFP transfusion in patients with DAPT. Furthermore, the incidence of reoperation for bleeding was significantly lower in TXA group patients who received DAPT or aspirin, compared with Control group. These clinical benefits may be more significant in patients with DAPT discontinued on the day of surgery.

The present study was also consistent with the recent systematic review highlighting the effectiveness of TXA in preventing APT-related bleeding (Fischer et al. 2020b). The prospective randomized studies (Myles et al. 2017, 2019) of patients undergoing CABG surgery with a two-by-two factorial design taking aspirin or placebo and tranexamic acid or placebo conducted by Myles et al. have revealed that TXA reduced post-operative bleeding risk, without increasing the risk of post-operative mortality or ischemic complications. In a recent study of 9535 patients undergoing noncardiac surgery, the incidence of the composite bleeding outcome was significantly lower with TXA than with placebo (Devereaux et al. 2022). A large retrospective cohort study including 19,687 patients who underwent off-pump CABG conducted by Wang et al. found that the application of TXA was safe and provided blood protection (Wang et al. 2022). Our result suggested that TXA administration effectively decreased post-operative bleeding and reduced the incidence of reoperations for bleeding and allogeneic blood transfusion in patients with DAPT preoperatively.

Subgroup analyses showed that studies with DAPT discontinued on the day of surgery significantly increased the risk of post-operative blood loss volume (*MD*: − 1.23 L vs. − 0.16 L) and post-operative RBC transfusion volume (*MD*: − 3.90 units vs. − 1.03 units) than those with DAPT discontinued less than 5–7 days preoperatively. Preoperative APT schemes are known to cause platelet dysfunction and are associated with increased risk of massive bleeding, blood transfusion, and complications (Valgimigli et al. 2018). The Society of Thoracic Surgeon/Society of Cardiovascular Anesthesiologists guidelines and European Association for Cardio-Thoracic Surgery (EACTS)/the European Association of Cardiothoracic Anaesthesiology (EACTA) guidelines have recommend that clopidogrel should be discontinued more than 3 days preceding surgery, and aspirin may be given the day before surgery (Society of Thoracic Surgeons Blood Conservation Guideline Task Force et al. 2011; Irving et al. 2020). The European Society of Cardiology (ESC) and the American Heart Association/American College of Cardiology guidelines recommend discontinuation of clopidogrel for at least 5 days (II A recommendation) to improve platelet function (Tibi et al. 2021). Despite the national/international guidelines or recommendations have documented antiplatelet regimens, the optimal perioperative management of patients with APT remains controversial. The decision-making of APT is complex and should be individually tailored to balance the risk of ischemia/thrombosis and bleeding (Irving et al. 2020; Tsan et al. 2023). The results of subgroup analyses demonstrated that the effectiveness of TXA in reducing bleeding may be more practical for patients with different DAPT discontinued time, especially for those with shorter withdrawal times. Therefore, further larger multicenter RCTs are needed to confirm our conclusions.

The present results demonstrated that the incidence of PC transfusion was 6.25%, and there was no significant difference between the groups. Although PC transfusion guidelines have been published, the transfusion practices were still heterogeneous (Yuan and Otrock 2021). TXA prevents the conversion of plasminogen to plasmin and preserves platelet functions (Klein et al. 2022). TXA may affect the interaction between glycoprotein Ib receptors and vWF and have positive impact on platelet functions to decrease the risk of bleeding (Mahla et al. 2018). For example, (Weber et al. 2011) proved that TXA administration was able to partially reverse platelet aggregation dysfunction induced by APT. Some other RCTs have also shown the effects of TXA in preventing blood loss in CABG patients on continuous aspirin therapy; nevertheless, TXA did not significantly lower the need for blood transfusions compared to control (Pleym et al. 2003; Guo et al. 2007). The pilot research conducted by Van Aelbrouck et al. has indicated that TXA treatment dramatically decreased the severity of CPB-induced platelet dysfunction in patients who were aspirin-free with normal preoperative platelet function (Aelbrouck et al. 2016). However, significant platelet dysfunction caused by preoperative administration of aspirin until the day of surgery was not improved by TXA administration in their study. The results from our meta-regression analysis of 842 patients did not find a correlation between the APT regimen (SAPT or DAPT) and the reduction of post-operative blood loss by TXA. The difference in the clinical trial designs, including aspirin withdraw time and different methods of parameters collection, may be contributed to the inconsistency of results.

In our meta-regression, results found that the TXA regimen (bolus or bolus plus continuous infusion) was not related to the reduction of post-operative blood loss by TXA. The strategy of TXA in cardiac surgery has long been a topic of debate. Fibrinolysis was inhibited by more than 90% at plasma concentrations of TXA approximately 20 µg/mL (Andersson et al. 1968). Some studies and reviews have found that effective plasma concentrations of TXA required to inhibit fibrinolysis in vitro was approximately 10 µg/mL in adults and 5 µg/mL in children (Picetti et al. 2019; Guo et al. 2019; Rozen et al. 2015; Grassin-Delyle et al. 2018). Another multicenter, double-blind, RCT published by (Shi et al. 2022), comparing the efficacy and adverse events of high-dose and low-dose TXA in cardiac surgical patients with CPB, has suggested that a high-dose regimen of TXA (30 mg/kg bolus and 16 mg/kg/h maintenance infusion) resulted in a statistically significant reduction in the incidence of allogeneic RBC transfusions. TXA administration varied significantly among studies, and there was no consensus on the ideal dosage of TXA, delivery methods (intravenous or topical), or continuous infusion/bolus regimen. Further trials should be conducted to verify the efficacy and optimal medication regimen of TXA administration for patients are at high-risk post-operative bleeding due to APT.

This study has some limitations. First, there were concerns with quality and heterogeneity of included studies, as well as heterogeneity in the various TXA regimens (e.g., medicine, dosage, route, time of administration), different surgical procedures (on-pump or off-pump), patient comorbidities, outcome definitions, and allogeneic transfusion protocols. Second, meta-analyses of pooled studies stratified by APT schemes and TXA regimens did not eliminate heterogeneity in our study outcomes. Different research designs, different methods of parameters collection, criteria for blood transfusion practices, and statistical analysis to determine the quality of the study could lead to heterogeneity between studies. Third, only 12 RCTs with comparatively small sample size were selected for the meta-analysis with respect to sample size, especially for several parameters, which may increase the chance of inaccurate conclusion and publication bias. We contacted the corresponding authors for missing data, but not much reply was received. Some recent studies have indicated that in a dose-dependent fashion, TXA was related to an increase of thrombotic complications (Tsan et al. 2023; Guo et al. 2019; Murkin et al. 2010). Therefore, the findings of the current study should be explained with caution. Large-scale randomized trials are warranted to make a strong recommendation for TXA in reversing bleeding related to APT.

## Conclusions

The current study provides some evidence that TXA is effective in reducing post-operative blood loss and allogeneic blood transfusion for adult cardiac surgical patients receiving preoperative APT. These potential clinical benefits may be greater in patients with aspirin, and clopidogrel continued closer to the day of surgery. Further, well-designed randomized trials are needed to confirm the effectiveness of TXA in improving platelet function.

### Supplementary Information

Supplementary Material 1. Supplemental Fig. 1. Risk of bias assessment summary.

Supplementary Material 2. Supplemental Fig. 2. Risk of bias assessment graph.

Supplementary Material 3. Supplemental Fig. 3. Forest plot of (A) platelet concentration transfusion volume and (B) transfusion rate.

Supplementary Material 4. Supplemental Fig. 4. Forest plot of (A) mechanical ventilation duration, (B) the length of stay in the intensive care unit, and (C) length of hospital stay.

Supplementary Material 5. Supplemental Fig. 5. Funnels plot examination for post-operative bleeding volume. 

Supplementary Material 6. Supplementary Table 1. PRISMA 2020 check lists.

Supplementary Material 7. Supplementary Table 2. Databases search strategies.

Supplementary Material 8. Supplemental Table 3. Subgroup analyses for the potential sources of heterogeneity.

Supplementary Material 9. Supplemental Table 4. Modified Jadad score of included studies.

Supplementary Material 10. Supplemental Table 5. Sensitivity analyses of high heterogeneity outcomes.

## Data Availability

No datasets were generated or analysed during the current study.

## Abbreviations

APT: Antiplatelet therapy
TXA: Tranexamic acid
CNKI: China National Knowledge Infrastructure
RCT: Randomized-controlled trial
RBC: Red blood cell
FFP: Fresh-frozen plasma
PC: Platelet concentrates
MVD: Mechanical ventilation duration
LOS: Length of stay
MD: Mean difference
CI: Confidence interval
OR: Odds ratio
DAPT: Dual antiplatelet treatment
CABG: Coronary artery bypass grafting
SAPT: Single antiplatelet treatment
IQR: Interquartile range
SD: Standard deviation
CPB: Cardiopulmonary bypass

## References

[CR1] Ahn SW, Shim JK, Youn YN, Song JW, Yang SY, Chung SC (2012). Effect of tranexamic acid on transfusion requirement in dual antiplatelet-treated anemic patients undergoing off-pump coronary artery bypass graft surgery. Circ J.

[CR2] Altun G, Hemşinli D, Pulathan Z, Civelek A (2017). Emergency coronary bypass surgery in patients under the influence of dual antiplatelet therapy: effects of tranexamic acid and desmopressin acetate. Turk J Med Sci.

[CR3] Amour J, Garnier M, Szymezak J, Le Manach Y, Helley D, Bertil S (2016). Prospective observational study of the effect of dual antiplatelet therapy with tranexamic acid treatment on platelet function and bleeding after cardiac surgery. Br J Anaesth.

[CR4] Andersson L, Nilsoon IM, Colleen S, Granstrand B, Melander B (1968). Role of urokinase and tissue activator in sustaining bleeding and the management thereof with EACA and AMCA. Ann N Y Acad Sci.

[CR5] Banihashem N, Khorasani M, Vaffai H, Naziri F, Khafri S, Seyfi S (2019). The effect of low- dose tranexamic acid on postoperative blood loss in patients treated with clopidogrel and aspirin. Caspian J Intern Med.

[CR6] Berger JS, Herout PM, Harshaw Q, Steinhubl SR, Frye CB, Becker RC (2012). Bleeding-associated outcomes with preoperative clopidogrel use in on- and off-pump coronary artery bypass. J Thromb Thrombolysis.

[CR7] Biancari F, Mariscalco G, Gherli R, Reichart D, Onorati F, Faggian G (2018). Variation in preoperative antithrombotic strategy, severe bleeding, and use of blood products in coronary artery bypass grafting: results from the multicentre E-CABG registry. Eur Heart J Qual Care Clin Outcomes.

[CR8] Devereaux PJ, Marcucci M, Painter TW, Conen D, Lomivorotov V, Sessler DI (2022). Tranexamic acid in patients undergoing noncardiac surgery. N Engl J Med.

[CR9] Egger M, Davey Smith G, Schneider M, Minder C (1997). Bias in meta-analysis detected by a simple, graphical test. BMJ.

[CR10] Fischer K, Awudi E, Varon J, Surani S (2020). Role of tranexamic acid in the clinical setting. Cureus.

[CR11] Fischer K, Bodalbhai F, Awudi E, Surani S (2020). Reversing bleeding associated with antiplatelet use: the role of tranexamic acid. Cureus.

[CR12] Grassin-Delyle S, Theusinger OM, Albrecht R, Mueller S, Spahn DR, Urien S (2018). Optimisation of the dosage of tranexamic acid in trauma patients with population pharmacokinetic analysis. Anaesthesia.

[CR13] Guo J, Gao X, Ma Y, Lv H, Hu W, Zhang S (2019). Different dose regimes and administration methods of tranexamic acid in cardiac surgery: a meta-analysis of randomized trials. BMC Anesthesiol.

[CR14] Guo Z, Jian K, Wei M, Guo Z, Li P, Han J (2007). Tranexamic acid and half dose aprotinin in off-pump coronary artery bypass. Chin Circ J.

[CR15] Hansson EC, Jidéus L, Åberg B, Bjursten H, Dreifaldt M, Holmgren A (2016). Coronary artery bypass grafting-related bleeding complications in patients treated with ticagrelor or clopidogrel: a nationwide study. Eur Heart J.

[CR16] Higgins JP, Altman DG, Gøtzsche PC, Jüni P, Moher D, Oxman AD (2011). The Cochrane Collaboration’s tool for assessing risk of bias in randomised trials. BMJ.

[CR17] Higgins JPT, Thomas J, Chandler J, Cumpston M, Li T, Page MJ, et al. Cochrane Handbook for Systematic Reviews of Interventions version 6.1. (updated September 2020). Cochrane, 2020. Available from www.training.cochrane.org/handbook.

[CR18] Irving AH, Harris A, Petrie D, Higgins A, Smith J, McQuilten ZK (2020). Impact of patient blood management guidelines on blood transfusions and patient outcomes during cardiac surgery. J Thorac Cardiovasc Surg.

[CR19] Jadad AR, Moore RA, Carroll D, Jenkinson C, Reynolds DJ, Gavaghan DJ (1996). Assessing the quality of reports of randomized clinical trials: is blinding necessary?. Control Clin Trials.

[CR20] Janssen PWA, Claassens DMF, Willemsen LM, Bergmeijer TO, Klein P, Ten Berg JM (2017). Perioperative management of antiplatelet treatment in patients undergoing isolated coronary artery bypass grafting in Dutch cardiothoracic centres. Neth Heart J.

[CR21] Khadanga P, Kanchi M, Gaur P (2020). Effectiveness of tranexamic acid in reducing postoperative blood loss in patients undergoing off-pump coronary artery bypass grafting. Cureus.

[CR22] Klein A, Agarwal S, Cholley B, Fassl J, Griffin M, Kaakinen T (2022). A review of European guidelines for patient blood management with a particular emphasis on antifibrinolytic drug administration for cardiac surgery. J Clin Anesth.

[CR23] Kremke M, Tang M, Bak M, Kristensen KL, Hindsholm K, Andreasen JJ (2013). Antiplatelet therapy at the time of coronary artery bypass grafting: a multicentre cohort study. Eur J Cardiothorac Surg.

[CR24] Landymore RW, Murphy JT, Lummis H, Carter C (1997). The use of low-dose aprotinin, epsilon-aminocaproic acid or tranexamic acid for prevention of mediastinal bleeding in patients receiving aspirin before coronary artery bypass operations. Eur J Cardiothorac Surg.

[CR25] Mahla E, Tantry US, Prüller F, Gurbel PA (2018). Is there a role for preoperative platelet function testing in patients undergoing cardiac surgery during antiplatelet therapy?. Circulation.

[CR26] Murkin JM, Falter F, Granton J, Young B, Burt C, Chu M (2010). High-dose tranexamic acid is associated with nonischemic clinical seizures in cardiac surgical patients. Anesth Analg.

[CR27] Murphy GJ, Reeves BC, Rogers CA, Rizvi SI, Culliford L, Angelini GD (2007). Increased mortality, postoperative morbidity, and cost after red blood cell transfusion in patients having cardiac surgery. Circulation.

[CR28] Myles PS, Smith JA, Forbes A, Silbert B, Jayarajah M, Painter T (2017). Tranexamic acid in patients undergoing coronary-artery surgery. N Engl J Med.

[CR29] Myles PS, Smith JA, Kasza J, Silbert B, Jayarajah M, Painter T (2019). Tranexamic acid in coronary artery surgery: one-year results of the Aspirin and Tranexamic Acid for Coronary Artery Surgery (ATACAS) trial. J Thorac Cardiovasc Surg.

[CR30] Pabinger I, Fries D, Schöchl H, Streif W, Toller W (2017). Tranexamic acid for treatment and prophylaxis of bleeding and hyperfibrinolysis. Wien Klin Wochenschr.

[CR31] Pagano D, Milojevic M, Meesters MI, Benedetto U, Bolliger D, von Heymann C (2018). 2017 EACTS/EACTA guidelines on patient blood management for adult cardiac surgery. Eur J Cardiothorac Surg.

[CR32] Paparella D, Brister SJ, Buchanan MR (2004). Coagulation disorders of cardiopulmonary bypass: a review. Intensive Care Med.

[CR33] Picetti R, Shakur-Still H, Medcalf RL, Standing JF, Roberts I (2019). What concentration of tranexamic acid is needed to inhibit fibrinolysis? A systematic review of pharmacodynamics studies. Blood Coagul Fibrinolysis.

[CR34] Pleym H, Stenseth R, Wahba A, Bjella L, Karevold A, Dale O (2003). Single-dose tranexamic acid reduces postoperative bleeding after coronary surgery in patients treated with aspirin until surgery. Anesth Analg.

[CR35] Rozen L, Faraoni D, Sanchez Torres C, Willems A, Noubouossie DC, Barglazan D (2015). Effective tranexamic acid concentration for 95% inhibition of tissue type plasminogen activator induced hyperfibrinolysis in children with congenital heart disease: a prospective, controlled, in-vitro study. Eur J Anaesthesiol.

[CR37] Shi J, Wang G, Lv H, Yuan S, Wang Y, Ji H, et al. Tranexamic acid in on-pump coronary artery bypass grafting without clopidogrel and aspirin cessation: randomized trial and 1-year follow-up. Ann Thorac Surg. 2013;95(3):795–802. 10.1016/j.athoracsur.2012.07.015 22959576

[CR38] Shi J, Wang Y, Xue Q, Yuan S, Wang G, Li L. Effectiveness and safety of tranexamic acid in patients receiving on-pump coronary artery bypass grafting without clopidogrel and aspirin cessation. Zhong Hua Wai Ke Za Zhi. 2013;51(6):527–32. 24091268

[CR36] Shi J, Ji H, Ren F, Wang G, Xu M, Xue Y, et al. Protective effects of tranexamic acid on clopidogrel before coronary artery bypass grafting: a multicenter randomized trial. JAMA Surg. 2013;148(6):538–47. 10.1001/jamasurg.2013.1560 23426385

[CR39] Shi J, Zhou C, Pan W, Sun H, Liu S, Feng W (2022). Effect of high- vs low-dose tranexamic acid infusion on need for red blood cell transfusion and adverse events in patients undergoing cardiac surgery: the OPTIMAL randomized clinical trial. JAMA.

[CR40] Ferraris VA, Brown JR, Despotis GJ, Hammon JW, Reece TB, Society of Thoracic Surgeons Blood Conservation Guideline Task Force (2011). update to the Society of Thoracic Surgeons and the Society of Cardiovascular Anesthesiologists blood conservation clinical practice guidelines. Ann Thorac Surg.

[CR41] Tibi P, McClure RS, Huang J, Baker RA, Fitzgerald D, Mazer CD (2021). STS/SCA/AMSECT/SABM update to the clinical practice guidelines on patient blood management. Ann Thorac Surg.

[CR42] Tomšič A, Schotborgh MA, Manshanden JS, Li WW, de Mol BA (2016). Coronary artery bypass grafting-related bleeding complications in patients treated with dual antiplatelet treatment. Eur J Cardiothorac Surg.

[CR43] Tsan SEH, Viknaswaran NL, Cheong CC, Cheah S, Ng KT, Mong SXY (2023). Prophylactic intravenous tranexamic acid and thromboembolism in non-cardiac surgery: a systematic review, meta-analysis and trial sequential analysis. Anaesthesia.

[CR44] Valgimigli M, Bueno H, Byrne RA, Collet JP, Costa F, Jeppsson A (2018). 2017 ESC focused update on dual antiplatelet therapy in coronary artery disease developed in collaboration with EACTS: the task force for dual antiplatelet therapy in coronary artery disease of the European Society of Cardiology (ESC) and of the European Association for Cardio-Thoracic Surgery (EACTS). Eur Heart J.

[CR45] Van Aelbrouck C, Jorquera-Vasquez S, Beukinga I, Pradier O, Ickx B, Barvais L (2016). Tranexamic acid decreases the magnitude of platelet dysfunction in aspirin-free patients undergoing cardiac surgery with cardiopulmonary bypass: a pilot study. Blood Coagul Fibrinolysis.

[CR46] Wan X, Wang W, Liu J, Tong T (2014). Estimating the sample mean and standard deviation from the sample size, median, range and/or interquartile range. BMC Med Res Methodol.

[CR47] Wang E, Yuan X, Wang Y, Chen W, Zhou X, Hu S (2022). Tranexamic acid administered during off-pump coronary artery bypass graft surgeries achieves good safety effects and hemostasis. Front Cardiovasc Med.

[CR48] Weber CF, Görlinger K, Byhahn C, Moritz A, Hanke AA, Zacharowski K (2011). Tranexamic acid partially improves platelet function in patients treated with dual antiplatelet therapy. Eur J Anaesthesiol.

[CR49] Yuan S, Otrock ZK (2021). Platelet transfusion: an update on indications and guidelines. Clin Lab Med.

