# Supplementary figures and images for: Hemostatic effects of tranexamic acid in cardiac surgical patients with antiplatelet therapy: a systematic review and meta-analysis
Source: Perioper Med (Lond). 2024 Jun 17;13:58. doi: 10.1186/s13741-024-00418-3 (PMC11184818; doi:10.1186/s13741-024-00418-3)

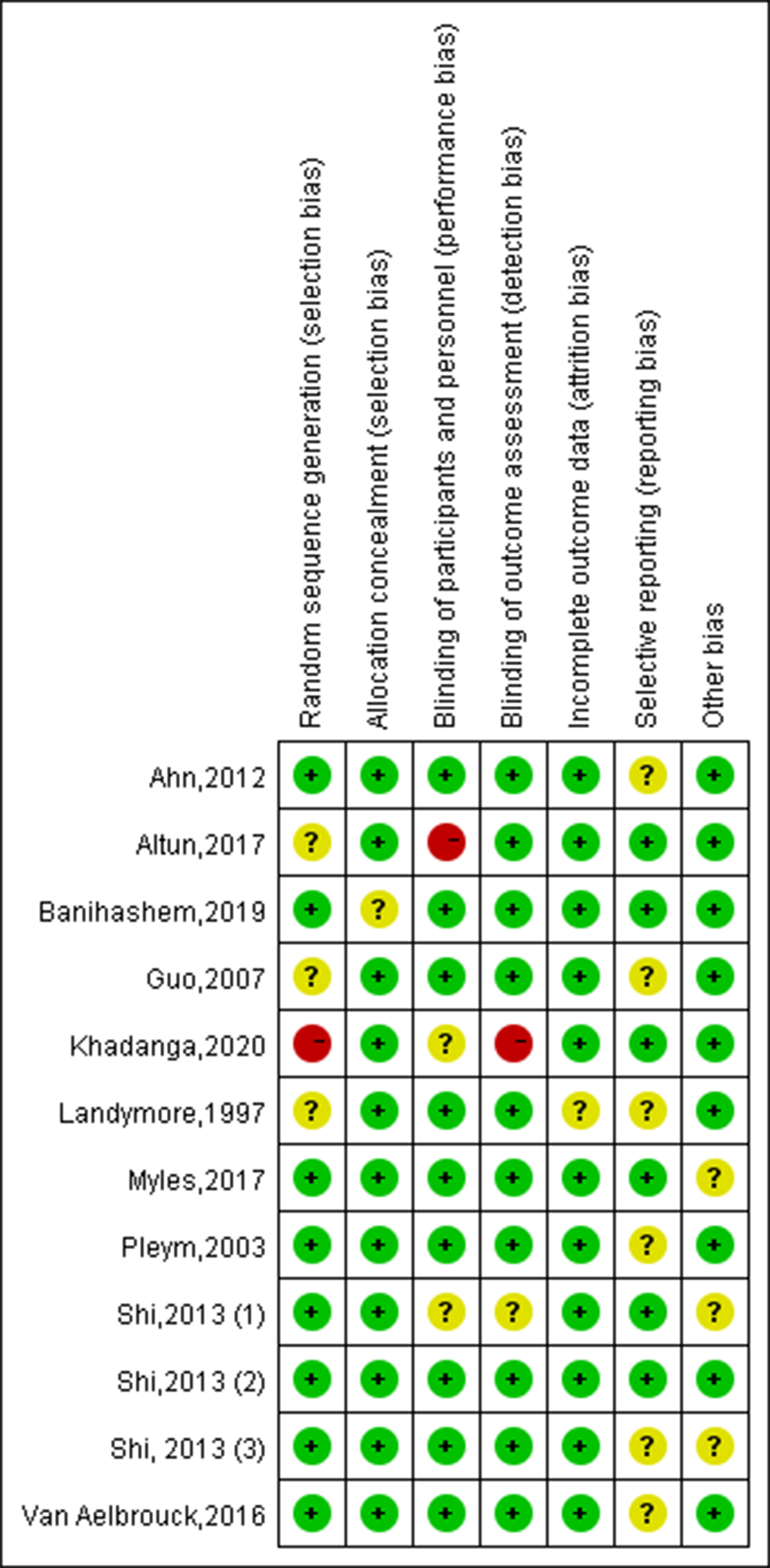

Supplement: Supplementary file 1 — Supplementary Material 1. Supplemental Fig. 1. Risk of bias assessment summary. [file 13741_2024_418_MOESM1_ESM.jpg]

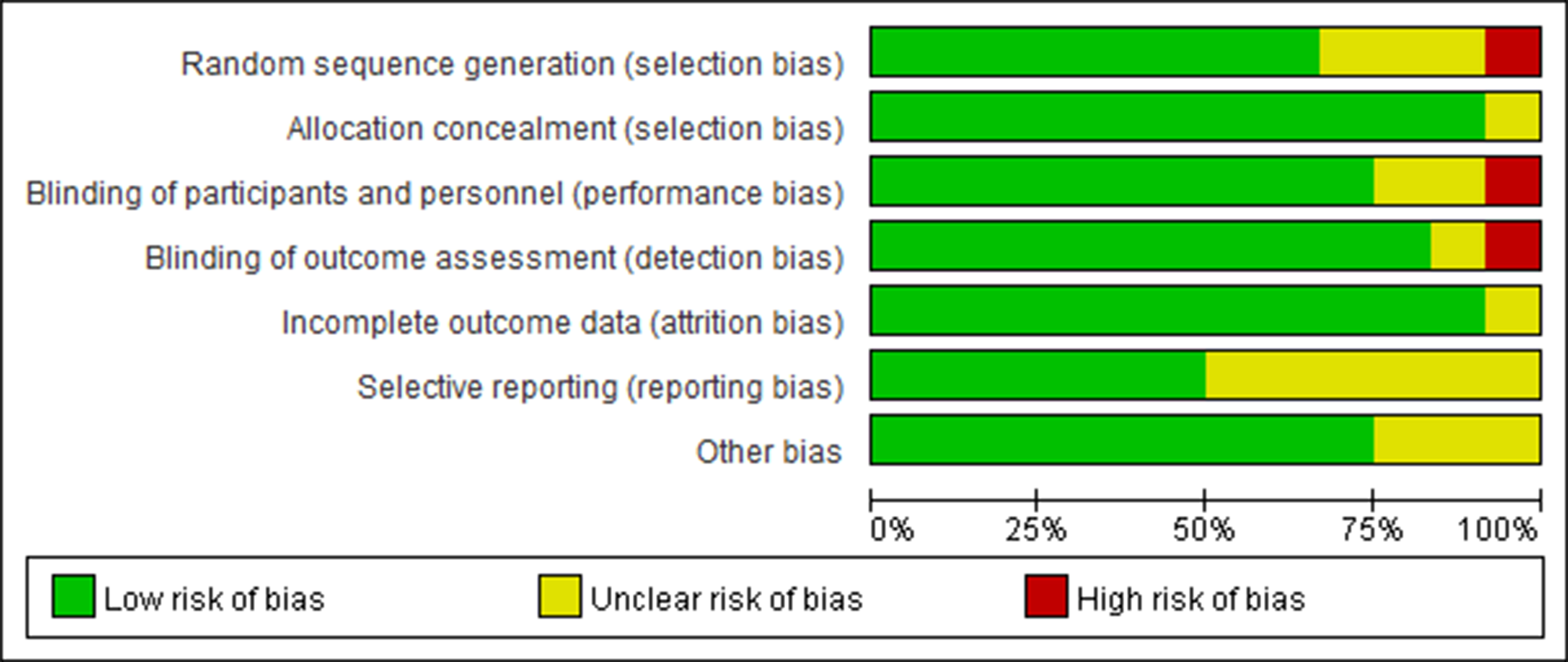

Supplement: Supplementary file 2 — Supplementary Material 2. Supplemental Fig. 2. Risk of bias assessment graph. [file 13741_2024_418_MOESM2_ESM.jpg]

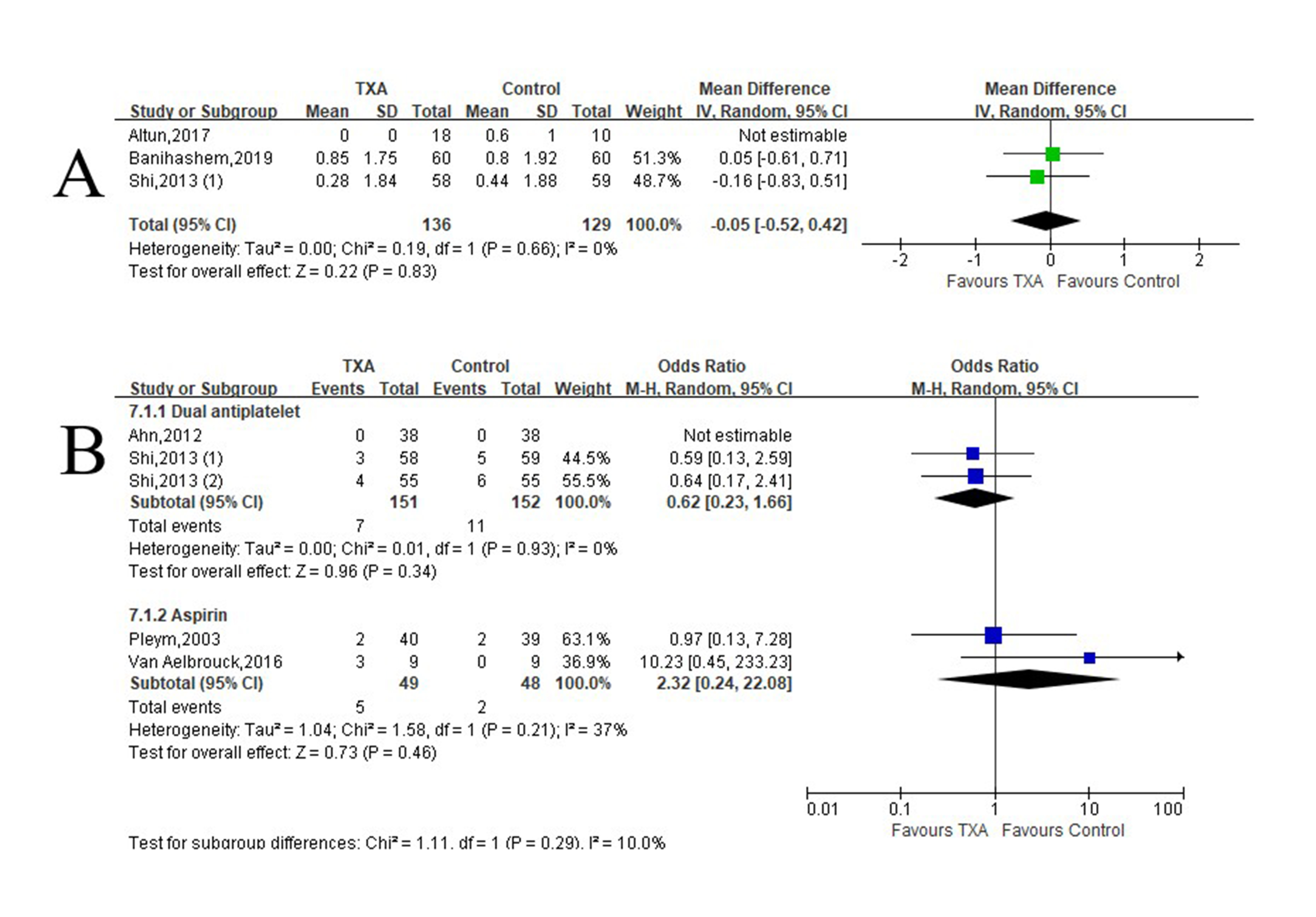

Supplement: Supplementary file 3 — Supplementary Material 3. Supplemental Fig. 3. Forest plot of (A) platelet concentration transfusion volume and (B) transfusion rate. [file 13741_2024_418_MOESM3_ESM.jpg]

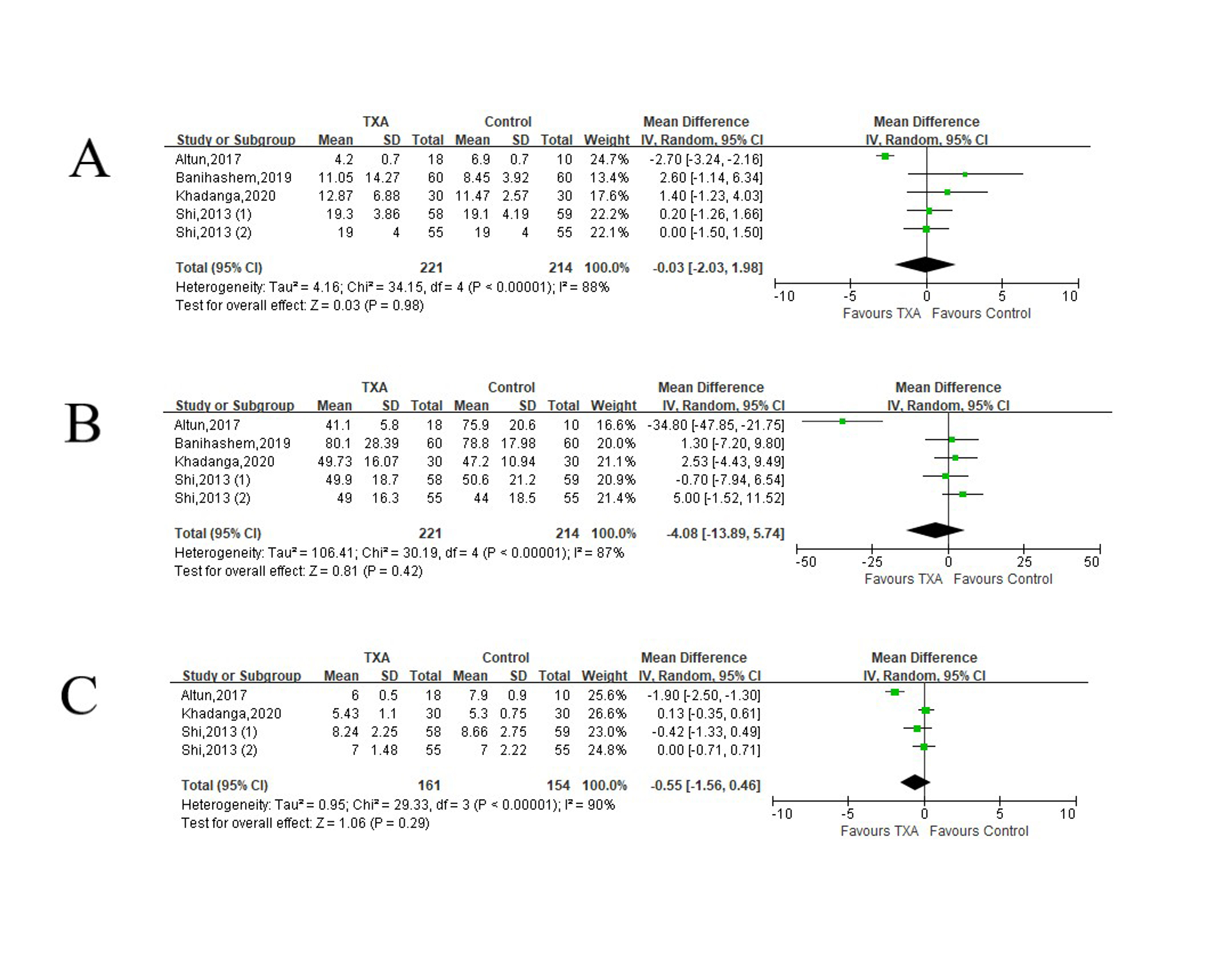

Supplement: Supplementary file 4 — Supplementary Material 4. Supplemental Fig. 4. Forest plot of (A) mechanical ventilation duration, (B) the length of stay in the intensive care unit, and (C) length of hospital stay. [file 13741_2024_418_MOESM4_ESM.jpg]

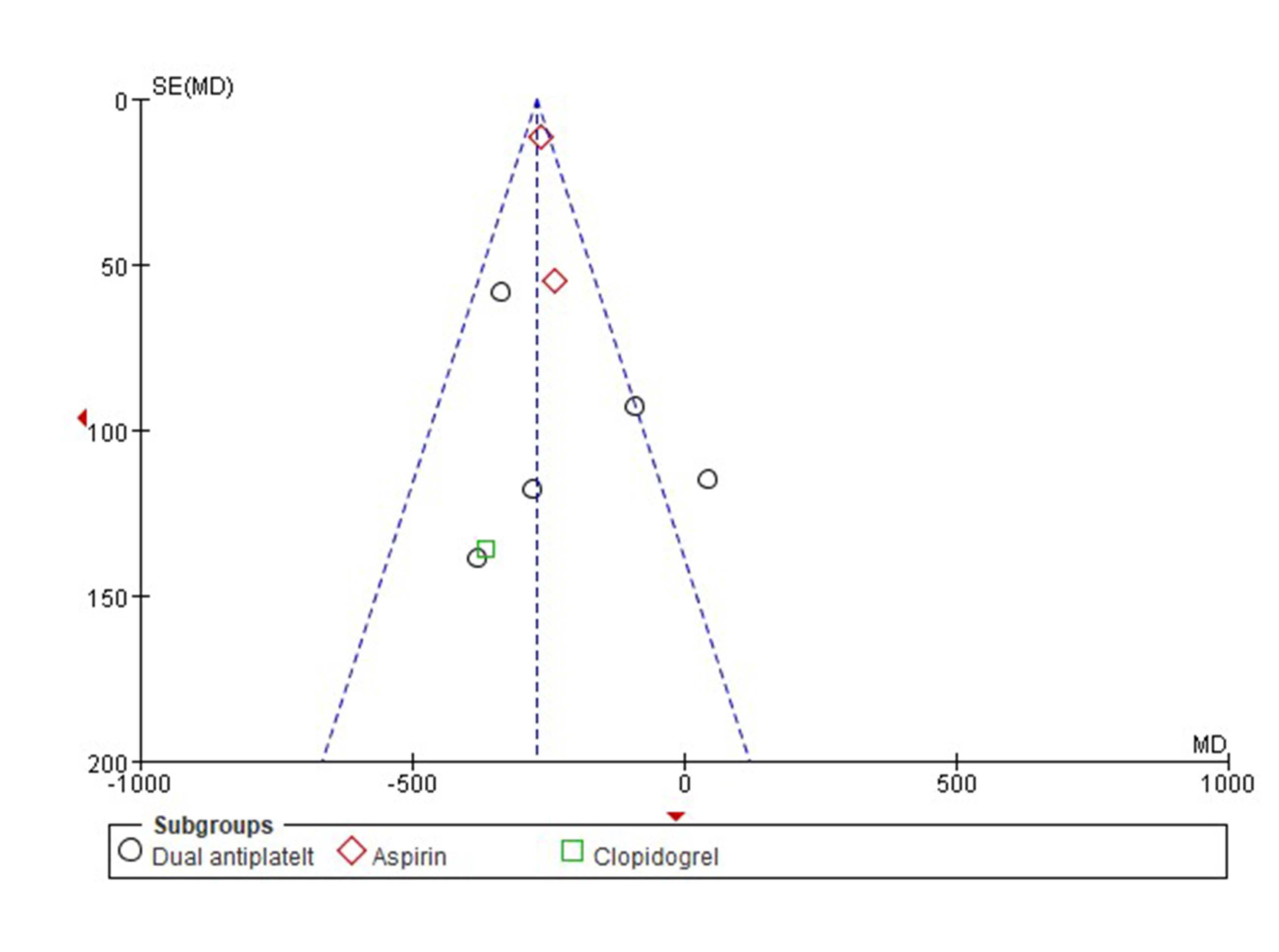

Supplement: Supplementary file 5 — Supplementary Material 5. Supplemental Fig. 5. Funnels plot examination for post-operative bleeding volume. [file 13741_2024_418_MOESM5_ESM.jpg]
