# Supplementary material for: Hemostatic effects of tranexamic acid in cardiac surgical patients with antiplatelet therapy: a systematic review and meta-analysis
Source: Perioper Med (Lond). 2024 Jun 17;13:58. doi: 10.1186/s13741-024-00418-3 (PMC11184818; doi:10.1186/s13741-024-00418-3)
Supplement: Supplementary file 7 — Supplementary Material 7. Supplementary Table 2. Databases search strategies. [file 13741_2024_418_MOESM7_ESM.docx]

**Supplementary Table 2. Databases search strategies**.

| **Search strategy** |  |  |
| --- | --- | --- |
| **PubMed** | Query | 88 |
| **#1** | "Tranexamic Acid"[Mesh] |  |
| **#2** | (((((coronary artery bypass surgery) OR (cardiac surgery)) OR (cardiovascular surgery)) OR (operation)) OR (procedure)) AND ("Tranexamic Acid"[Mesh]) |  |
| **#3** | ((((((((aspirin) OR (clopidogrel)) OR (antiplatelet)) OR (single antiplatelet therapy)) OR (dual antiplatelet therapy)) OR (SAPT)) OR (DAPT)) OR (Acetylsalicylic Acid) OR (ticagrelor))) AND ((((((coronary artery bypass surgery) OR (cardiac surgery)) OR (cardiovascular surgery)) OR (operation)) OR (procedure)) AND ("Tranexamic Acid"[Mesh])) |  |
| **OVID** |  | 85 |
| **#1** | coronary artery bypass surgery or cardiac surgery or cardiovascular surgery or procedure or operation |  |
| **#2** | Tranexamic Acid |  |
| **#3** | aspirin or clopidogrel or antiplatelet or single antiplatelet therapy or dual antiplatelet therapy or SAPT or DAPT or Acetylsalicylic Acid or ticagrelor |  |
| **#4** | #1 and #2 and #3 |  |
| **#5** | controlled clinical trial or randomly or placebo |  |
| **#6** | #1 and #2 and #3 and #5 |  |
| **Embase** |  | 139 |
|  | (‘coronary artery bypass surgery’: ti,ab,kw OR ‘cardiac surgery’:ti,ab,kw OR ‘cardiovascular surgery’: ti,ab,kw OR ‘procedure’: ti,ab,kw OR ‘operation’: ti,ab,kw’) AND ‘tranexamic acid’: ti,ab,kw; AND (aspirin’: ti,ab,kw OR ‘clopidogrel’: ti,ab,kw OR ‘antiplatelet’:ti,ab,kw OR ‘single antiplatelet therapy’:ti,ab,kw OR ‘dual antiplatelet therapy’:ti,ab,kw OR ‘SAPT’:ti,ab,kw OR ‘DAPT’:ti,ab,kw OR ‘Acetylsalicylic Acid’:ti,ab,kw OR ‘ticagrelor’:ti,ab,kw) AND or (‘trial’:ti,ab,kw OR ‘randomly’:ti,ab,kw OR ‘placebo’:ti,ab,kw) |  |
| **Cochrane** |  | 56 |
| **#1** | (coronary artery bypass surgery):ti,ab,kw OR (cardiac surgery):ti,ab,kw OR (cardiovascular surgery):ti,ab,kw OR (procedure):ti,ab,kw OR (operation):ti,ab,kw |  |
| **#2** | (Tranexamic Acid):ti,ab,kw |  |
| **#3** | (aspirin):ti,ab,kw OR (clopidogrel):ti,ab,kw OR (antiplatelet):ti,ab,kw OR (single antiplatelet therapy):ti,ab,kw OR (dual antiplatelet therapy):ti,ab,kw OR (SAPT):ti,ab,kw OR (DAPT):ti,ab,kw OR (Acetylsalicylic Acid):ti,ab,kw OR (ticagrelor):ti,ab,kw |  |
| **#4** | (trial):ti,ab,kw OR (randomly):ti,ab,kw OR (placebo):ti,ab,kw |  |
| **#5** | #1 and #2 and #3 and #4 |  |
| **CNKI** |  | 50 |
|  | ((SU = 心脏手术) OR (KW = 心脏手术) OR (SU= 瓣膜置换术) OR (KW =瓣膜置换术) OR (SU =心脏不停跳手术) OR (KW =心脏不停跳手术) OR (SU =冠脉搭桥手术) OR (KW =冠脉搭桥手术) OR (SU =心脏外科手术) OR (KW =心脏外科手术)) AND ((SU = 抗血小板治疗) OR (KW =抗血小板治疗) OR (SU = 阿司匹林) OR (KW =阿司匹林) OR (SU = 阿司匹林肠溶片) OR (KW =阿司匹林肠溶片) OR (SU = 氯吡格雷) OR (KW =氯吡格雷) OR (SU = 替格瑞洛) OR (KW =替格瑞洛)) AND ((SU = 氨甲环酸) OR (KW = 氨甲环酸)) |  |
