# Supplementary material for: Hemostatic effects of tranexamic acid in cardiac surgical patients with antiplatelet therapy: a systematic review and meta-analysis
Source: Perioper Med (Lond). 2024 Jun 17;13:58. doi: 10.1186/s13741-024-00418-3 (PMC11184818; doi:10.1186/s13741-024-00418-3)
Supplement: Supplementary file 8 — Supplementary Material 8. Supplemental Table 3. Subgroup analyses for the potential sources of heterogeneity. [file 13741_2024_418_MOESM8_ESM.docx]

Supplemental Table 3**.** Subgroup analyses for the potential sources of heterogeneity

| Variables | Outcomes | No. of Comparisons | WMD | 95% CI | Heterogeneity *P* | *I^2^* | Overall effect *P* | *P_Difference_* |
| --- | --- | --- | --- | --- | --- | --- | --- | --- |
| **1. DAPT withdrawal time** | Post-op bleeding  (mL) | 5 |  |  |  |  |  | < 0.00001 |
| Withdrawal on the day of surgery |  | 1 | -1232.00 | -1421.57, -1042.43 | - | - | < 0.00001 |  |
| Withdrawal less than 5-7 days preoperatively |  | 4 | -154.91 | -265.22, -44.61 | 0.09 | 54% | 0.006 |  |
| **2. DAPT withdrawal time** | Post-op RBC  (U) | 5 |  |  |  |  |  | < 0.00001 |
| Withdrawal on the day of surgery |  | 1 | -3.90 | -4.75, -3.05 | - | - | < 0.00001 |  |
| Withdrawal less than 5-7 days preoperatively |  | 4 | -1.03 | -1.96, -0.10 | 0.05 | 61% | 0.03 |  |
| **3. DAPT withdrawal time** | Post-op FFP  (U) | 4 |  |  |  |  |  | 0.0002 |
| Withdrawal on the day of surgery |  | 1 | -3.30 | -4.24, -2.36 | - | - | < 0.00001 |  |
| Withdrawal less than 5-7 days preoperatively |  | 3 | -0.89 | -1.72, -0.05 | 0.04 | 68% | 0.04 |  |
| **4. CPB** | Post-op bleeding  (mL) | 6 |  |  |  |  |  | 0.33 |
| Off-pump |  | 2 | -172.21 | -520.86, 176.44 | 0.006 | 87% | 0.33 |  |
| On-pump |  | 4 | -497.08 | -1052.15, 57.99 | < 0.00001 | 96% | 0.08 |  |
| **5. CPB** | Post-op RBC  (U) | 5 |  |  |  |  |  | 0.09 |
| Off-pump |  | 1 | -0.60 | -1.06, -0.14 |  |  | 0.01 |  |
| On-pump |  | 4 | -2.54 | -4.76, -0.33 | < 0.00001 | 91% | 0.02 |  |
| **6. CPB** | Post-op FFP  (U) | 4 |  |  |  |  |  | 0.03 |
| Off-pump |  | 1 | -0.34 | -0.67, -0.01 |  |  | 0.04 |  |
| On-pump |  | 3 | -2.09 | -3.59, -0.58 | 0.002 | 84% | 0.007 |  |

DAPT = dual antiplatelet therapy, CPB = cardiopulmonary bypass, Post-op = postoperative, RBC = red blood cell, FFP = fresh frozen plasma, TXA = tranexamic acid, WMD = weighted mean difference, CI = confidence interval
