# Supplementary material for: Hemostatic effects of tranexamic acid in cardiac surgical patients with antiplatelet therapy: a systematic review and meta-analysis
Source: Perioper Med (Lond). 2024 Jun 17;13:58. doi: 10.1186/s13741-024-00418-3 (PMC11184818; doi:10.1186/s13741-024-00418-3)
Supplement: Supplementary file 9 — Supplementary Material 9. Supplemental Table 4. Modified Jadad score of included studies. [file 13741_2024_418_MOESM9_ESM.docx]

Supplemental Table 4. Modified Jadad score of included studies.

| Study | Sample size | Modified Jadad score | | | | |
| --- | --- | --- | --- | --- | --- | --- |
|  |  | Randomization | Concealment of allocation | Double blinding | Withdrawals and dropouts | Total |
| Ahn,2012 [23] | 76 | 2 | 2 | 2 | 1 | 7 |
| Shi,2013 (1) [24] | 117 | 2 | 2 | 2 | 1 | 7 |
| Shi,2013 (2) [25] | 110 | 2 | 2 | 2 | 1 | 7 |
| Altun,2017 [26] | 54 | 1 | 0 | 0 | 1 | 2 |
| Banihashem,2019 [27] | 120 | 2 | 2 | 2 | 0 | 6 |
| Khadanga,2020 [28] | 60 | 1 | 0 | 0 | 0 | 1 |
| Landymore,1997 [29] | 198 | 1 | 0 | 2 | 0 | 3 |
| Pleym,2003 [30] | 79 | 2 | 1 | 2 | 1 | 6 |
| Guo,2007 [31] | 112 | 1 | 0 | 2 | 1 | 4 |
| Van Aelbrouck,2016 [32] | 28 | 2 | 2 | 1 | 1 | 6 |
| Myles,2017 [33] | 4662 | 2 | 2 | 2 | 1 | 7 |
| Shi,2013 (4) [34] | 552 | 2 | 2 | 2 | 1 | 7 |
