# Supplementary material for: Hemostatic effects of tranexamic acid in cardiac surgical patients with antiplatelet therapy: a systematic review and meta-analysis
Source: Perioper Med (Lond). 2024 Jun 17;13:58. doi: 10.1186/s13741-024-00418-3 (PMC11184818; doi:10.1186/s13741-024-00418-3)
Supplement: Supplementary file 10 — Supplementary Material 10. Supplemental Table 5. Sensitivity analyses of high heterogeneity outcomes. [file 13741_2024_418_MOESM10_ESM.docx]

Supplemental Table 5**.** Sensitivity analyses of high heterogeneity outcomes.

| Heterogeneity  outcome | Excluded trials | TXA  (n) | Control  (n) | Heterogeneity  *I*^2^ (%) *P* | | Analysis model | WMD | 95% CI | Overall effect *P* |
| --- | --- | --- | --- | --- | --- | --- | --- | --- | --- |
| **DAPT** | | | | | | | | | |
| Post-op bleeding (mL) | [23], [26] | 203 | 204 | 47 | 0.13 | IV Fix | -279.96 | (-364.65, -195.27) | ＜0.00001 |
| Post-op RBC (U) | [24], [26] | 123 | 123 | 14 | 0.31 | IV Fix | -0.62 | (-1.02, -0.22) | 0.002 |
| Post-op FFP (U) | [23], [26] | 113 | 114 | 21 | 0.26 | IV Fix | -1.27 | (-2.02, -0.52) | 0.0009 |
| MVD (hours) | [26] | 203 | 204 | 0 | 0.53 | IV Fix | 0.43 | (-0.51, 1.36) | 0.37 |
| ICU stay (hours) | [26] | 203 | 204 | 0 | 0.71 | IV Fix | 2.26 | (-1.34, 5.87) | 0.22 |
| Hospital length of stay (days) | [26] | 143 | 144 | 0 | 0.58 | IV Fix | 0.01 | (-0.35, 0.37) | 0.96 |
| **Aspirin** | | | | | | | | | |
| Post-op RBC(U) | [31], [33] | 96 | 89 | 0 | 0.4 | IV Fix | -1.30 | (-1.45, -1.16) | ＜ 0.00001 |

DAPT = dual antiplatelet therapy, Post-op = postoperative, RBC = red blood cell, FFP = fresh frozen plasma, MVD= mechanical ventilation duration, ICU = intensive care unit, TXA = tranexamic acid, WMD = weighted mean difference, CI = confidence interval
